# Key Adaptive Trait Promotes Contrasting Modes of Diversification in a Bivalve Clade

**DOI:** 10.1007/s11692-024-09643-6

**Published:** 2024-11-28

**Authors:** Emily R. Nigro, Katie S. Collins, Stewart M. Edie, Nicholas M. A. Crouch, David Jablonski

**Affiliations:** 1 Department of the Geophysical Sciences, University of Chicago, 5734 South Ellis Ave, Chicago, IL 60637 USA; 2 Earth and Planetary Sciences Department, Stanford University, Stanford, CA 94305 USA; 3 Natural History Museum, London, SW7 5BD UK; 4 Department of Paleobiology, National Museum of Natural History, Smithsonian Institution, Washington, DC 20013 USA; 5 Committee on Evolutionary Biology, University of Chicago, Chicago, IL 60637 USA

**Keywords:** Adaptation, Diversification, Bivalvia, Functional morphology, Key traits

## Abstract

Siphons in bivalves have been postulated as a key adaptive trait, enabling modes of life inaccessible to asiphonate lineages, that afford better protection from predation and dislodgement, thereby enhancing their taxonomic diversification. To test the impact of siphons on diversity, we compared two bivalve clades with similar shell forms and life positions that differ in the presence/absence of this supposed key trait: the asiphonate Archiheterodonta (origin ~ 420 Myr ago) and the siphonate Veneridae (origin ~ 170 Myr ago). We measured three characters relevant to burrowing (shell length, cross-sectional area, and proportional shell volume) in these two groups, finding that siphonate venerids occupy more modes of life than archiheterodonts because they can live at a greater range of distances from the sediment–water interface, with the thinnest shells occurring in the deepest-burrowing groups. Asiphonate taxa have thicker shells, perhaps as a compensatory adaptation in response to the potential for exposure and attack because they are limited to shallower depths of burial. The lack of siphons may have impeded morphologic and taxonomic diversification in archiheterodonts. In contrast, siphons are consistent with a key adaptive trait in the Veneridae, evidently enabling taxonomic diversification into a greater range of morphologies.

**Supplementary Information:**

The online version contains supplementary material available at 10.1007/s11692-024-09643-6.

## Introduction

The evolution of a particular adaptive trait or traits is often held as key to the radiation of certain groups [e.g. floral nectar spurs in columbines, C4 photosynthesis in monocots, image-forming eyes in metazoans, pharyngeal jaws in labroid fishes (de Queiroz, 2002; Miller et al., 2023), and a general summary in Rabosky, 2017], but the unambiguous identification of such traits has proven difficult (Erwin, 2021; Jablonski, 2022; Miller et al., 2023). A key trait may release a clade from evolutionary constraints, either intrinsic (e.g. developmental) or extrinsic (e.g. environmental), providing an opportunity for diversification. The expression of other characters may be impacted by the key trait, allowing for stronger selection on other aspects of the organism's phenotype and thus influence the clade’s taxonomic diversification. Key traits may also be interpreted as innovations that promote an ecological shift, allowing a clade to exploit a novel mode of life (Jablonski, 2022; Miller & Stroud, 2021), but in this study we consider them to be characters imparting increased diversification regardless of whether or not an ecological shift occurs. Assessing whether a putative key trait is actually crucial to clade radiation requires context: comparisons of rates and patterns of diversification between clades that are similar except for presence or absence of the supposed key trait. Here, we use additional macroevolutionary currencies—functional variety and morphological disparity—to tease apart the impacts of a key trait on the differential dynamics of taxonomic richness between two major groups of marine bivalves.

### Siphons as a Putative Key Trait

**Fig. 1:**
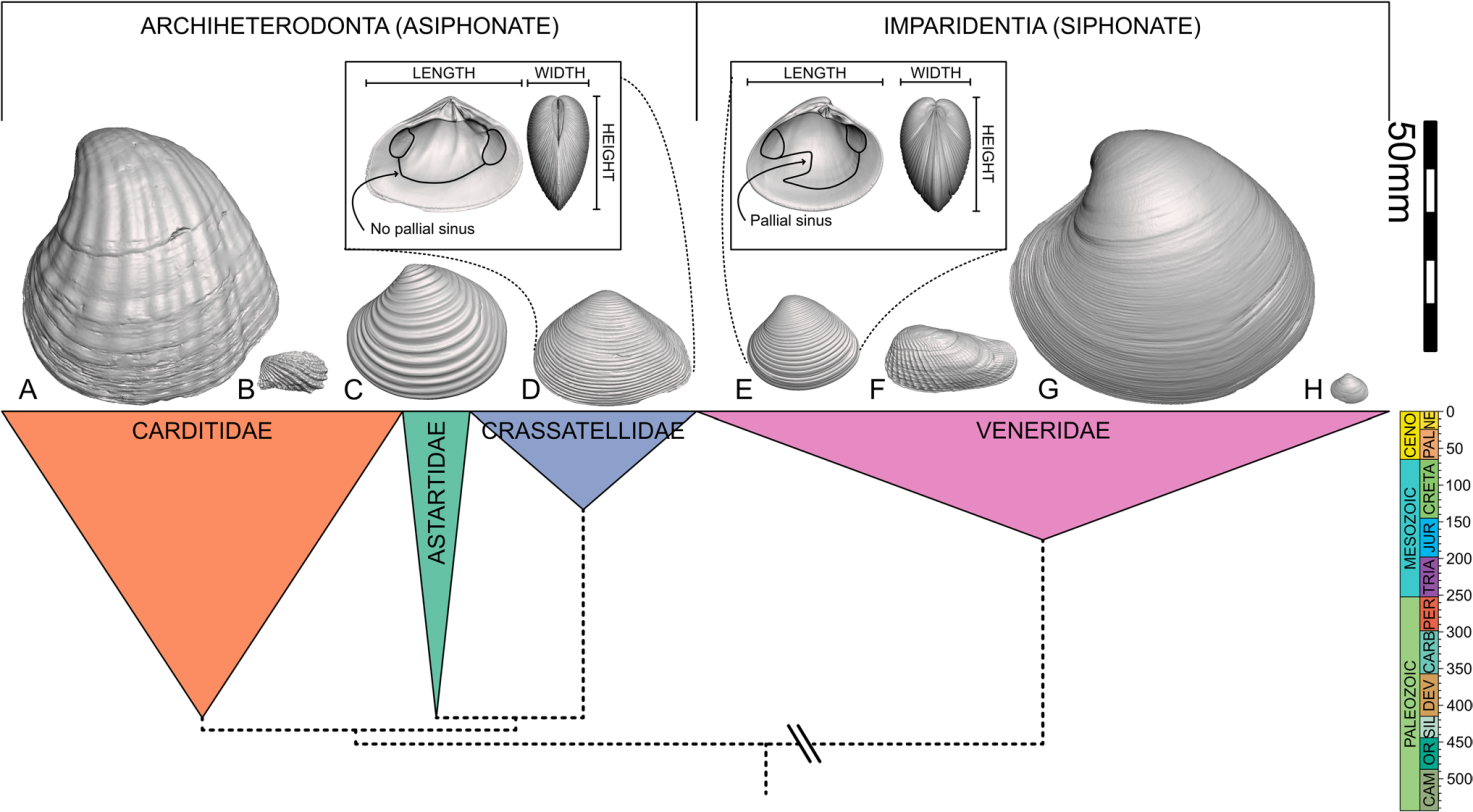
Phylogeny of families included in this study, with exemplar species for each substratum group depicted to scale (left valve exteriors only). Height of colored triangles corresponds to the geological age of clade (timescale on the right). Width of colored triangle corresponds to number of species included in this study. Node heights are not timescaled. Exemplar taxa showcase the range of morphologies and functions found in the dataset. **A** *Strophocardia megastropha* (Gray, 1825) (Carditidae; infaunal asiphonate). **B** *Cardita calyculata* (Linnaeus, 1758) (Carditidae; epifaunal). **C** *Astarte sulcata* (da Costa, 1778) (Astartidae; infaunal asiphonate). **D** *Crassatella capensis* Lamy, 1917 (Crassatellidae; infaunal asiphonate). **E** *Transenpitar americana* (Doello-Jurado, 1951) (Veneridae; shallow infaunal siphonate). **F** *Petricolaria pholadiformis* (Lamarck, 1815) (Veneridae; endolithic). **G** *Agriopoma catharia* (Dall, 1902) (Veneridae; deep infaunal siphonate). **H** *Cooperella atlantica* Rehder, 1943 (Veneridae; nestler). Scale bar (top right) is 50 mm, and applies to all unannotated shell images (**A**–**H**). Additional annotated images (not to scale) are provided of *Crassatella capensis* and *Agriopoma catharia* showing the presence/absence and position of the pallial sinus, and the usage of ‘length’, ‘height’, and ‘width’ in this study

The molluscan Class Bivalvia provides a rich dataset for testing the links between hypothesized key traits and diversifications, with its fossil record spanning > 500 million years, and global coverage in marine and freshwater deposits. Bivalves’ calcium carbonate shells, which fossilize easily and frequently, contain extensive morphological data that can be linked to function and reveal basic details of soft-part anatomy such as the presence or absence of the soft-tissue structures known as siphons, inferred from the presence or absence of a pallial sinus impressed on the inner surface of the shell (Fig. 1). Siphons are fused layers of the mantle that form a tube or pair of tubes analogous to a snorkel; this allows the inhalant current to bring water into the body to pass over the gills for respiration and often for suspension-feeding, and then exit the body carrying waste via the exhalant current. Siphons have been proposed as a key trait promoting the prolific diversification of the infaunal siphonate taxa (Stanley, 1968). Siphons are, of course, not the only trait relevant to bivalve life habits, but the acquisition of siphons certainly enables additional modes of exploitation of substrata and food supplies, for example, they can enable faster burrowing (via increased hydraulic pressure and water pumping), exploitation of additional habitats (e.g. deeper burrowing and rock boring) and they also allow entirely new forms of feeding, such as feeding on detritus on the sediment surface (e.g. in Tellinidae [Stanley, 1968; Yonge, 1949]), ectoparasitism (e.g. in Galeommatoidea [Oliver & Lützen, 2011]), and carnivory (e.g. in Cuspidariidae [Morton & Machado, 2019; Yonge, 1928]). Further, they are among the few functionally important traits not shared by our two target clades.

Proximity to the sediment–water interface brings bivalves into closer contact with potential predators and makes them more likely to be dislodged or covered with sediment by wave energy if they are nearshore. Asiphonate infaunal bivalves must live within a few mm of the sediment–water interface (Stanley, 1968). Siphons allow the animal to position itself further from the sediment–water interface (regardless of which substrate it occupies) than an asiphonate individual can, where it will be safer from predation and disturbance. Pallial sinus dimensions approximate the absolute size of siphons and habitual burrowing depth (Kondo, 1987), and broadly reflect potential burial depth, in that a very shallow pallial sinus cannot house large siphons; taxa with shallow sinuses do not burrow deeply, even though taxa with large pallial sinuses may live very shallowly. Siphonate bivalves do not entirely escape from predators, such as crabs and burrowing snails (Dudas et al., 2005; Haddon et al., 1987; Visaggi et al., 2013; Whitlow, 2010), but increased burial depth does provide protection, with fewer bivalves eaten from deeper sediments in experimental studies (e.g. Dudas et al., 2005; Haddon et al., 1987). The argument has been made that reduced pressure from predation and abiotic disturbance—specifically owing to the evolution of siphons—has promoted extensive diversification of siphonate relative to asiphonate infaunal clades (e.g. Stanley, 1968, 2015a).

### Comparative Testing of Siphons as a Key Trait

We focus on two groups of bivalves which are similar in terms of habitats, distribution, and relevant functional morphology (all being proso- or opisthogyrous, equivalve, and eulamellibranch). The earliest bivalves were asiphonate (Stanley, 1968, 2015b), and either epifaunal or shallow burrowing (Fang & Sanchez, 2012; Stanley, 2015b). Asiphonate burrowing heterodont taxa still exist as the ancient clades Palaeoheterodonta and Archiheterodonta (Table 1). The single living genus of marine Palaeoheterodonta, *Neotrigonia*, has filibranch rather than eulamellibranch gills and is excluded from this analysis. The Archiheterodonta are eulamellibranch, asiphonate suspension-feeding bivalves, containing the families Astartidae, Carditidae, Crassatellidae, and the minute “Condylocardiidae” [included within Carditidae herein following González and Giribet (2015) and Dias Passos et al. (2021)]. They are primarily shallow-infaunal, occasionally epifaunal. The archiheterodonts are sister to the largest clade of Bivalvia, the siphonate Euheterodonta, which contains > 2300 fossil and living genera in the two clades Imparidentia and Anomalodesmata (Foote et al., 2024; Huang et al., 2023) (Table 1), and exhibits a remarkable range of morphologies and life habits. We exclude the Anomalodesmata as many of their disparate morphologies and ecologies have no equivalent within the archiheterodonts, and focus here on only the most speciose family (over 700 extant species [Huang et al., 2023]) within the Imparidentia, the Veneridae (Stanley, 1968. Venerids are the euheterodont bivalves that most closely resemble archiheterodonts in shell form, gill structure, foot behavior, and modes of life. Like archiheterodonts, venerids are also exclusively eulamellibranch suspension-feeders, and primarily but not exclusively infaunal.

**Table 1:** Taxon counts for siphonate and non-siphonate heterodont clades, extinct and modern marine (excludes freshwater taxa)

Clade	Siphonate	Fossil genera Foote et al., (2024)*	Extant genera [species] Huang et al., (2023)	Extant genera [species] in this study	% of extant genera used in this study
Palaeoheterodonta	No	201	1 [8]	0 [0]	0
Archiheterodonta	No	203	72 [397]	45 [124]	62%
Astartidae	No	–	7 [38]	3 [12]	43%
Carditidae	No	–	48 [270]	28 [72]	58%
Crassatellidae	No	–	17 [89]	14 [40]	82%
Anomalodesmata	Yes	218	87 [460]	0 [0]	0
Imparidentia	Yes	1420	647 [3199]	124 [126]	19%
Veneridae	Yes	–	138 [745]	124 [126]	90%

Recent taxon counts are from Huang et al., (2023), fossil taxon counts from Foote et al., (2024). Siphonate genera (n = 734 extant and 1638 fossil) are almost four times as numerous as asiphonate taxa (n = 73 extant and 404 fossil)

*Note that Foote et al. do not provide a breakdown of family-level assignments of genera in their archiheterodont dataset

The diversity contrast between Archiheterodonta (397 species) and Veneridae (745 species) is particularly striking given that the former are much older (~ 420 Myr vs ~ 170 Myr: Crouch et al., 2021) and that they started the Cenozoic with half as much diversity [6 archiheterodonts are present in the Danian as opposed to 3 venerids (Edie et al., 2018 supplementary material)]. Many venerids live just below the sediment water-interface—similar in life position and feeding habit to the archiheterodonts, making both groups vulnerable to the same broad pressures and threats, but other venerids are deeper burrowers, or rock-borers and nestlers in hard substrata.

Foot anatomy of both clades is broadly similar: Saleuddin (1965)’s work on *Astarte* (Astartidae), and Park et al. (2012) on *Gomphina veneriformis* (Veneridae) both found that the foot consists of an epithelial layer, bands of muscle, and connective tissue. In general, the foot of shallow infaunal siphonate, and infaunal asiphonate, groups is described as ‘bladelike’ or ‘hatchet-shaped’ (Kranz, 1974). Behavioral studies are few but have shown that both archiheterodonts (Saleuddin, 1965; Stanley, 1970; Harry, 1966; Ansell, 1969) and venerids (Stanley, 1970, Ansell, 1969, Hornell, 1917) are capable of crawling and leaping, at least in captivity. Archiheterodonts are sluggish burrowers in general but venerids may be sluggish or fast (Stanley, 1970). Burrowing speed, however, is attributed by authors (e.g. Stanley, 1970) more to shell morphology (e.g. thickness, sculpture) than to the foot.

Archiheterodonts and venerids are equivalve, generally either equilateral or posteriorly elongate to some extent, and proso- or orthogyrous in their coiling geometry, meaning their shell forms are broadly similar and can be effectively compared (Fig. 1). Venerids are primitively siphonate, hypothesized to have split from the extinct, siphonate Family Isocyprinidae in the mid-Jurassic Period (Gardner, 2005).

**Fig. 2:**
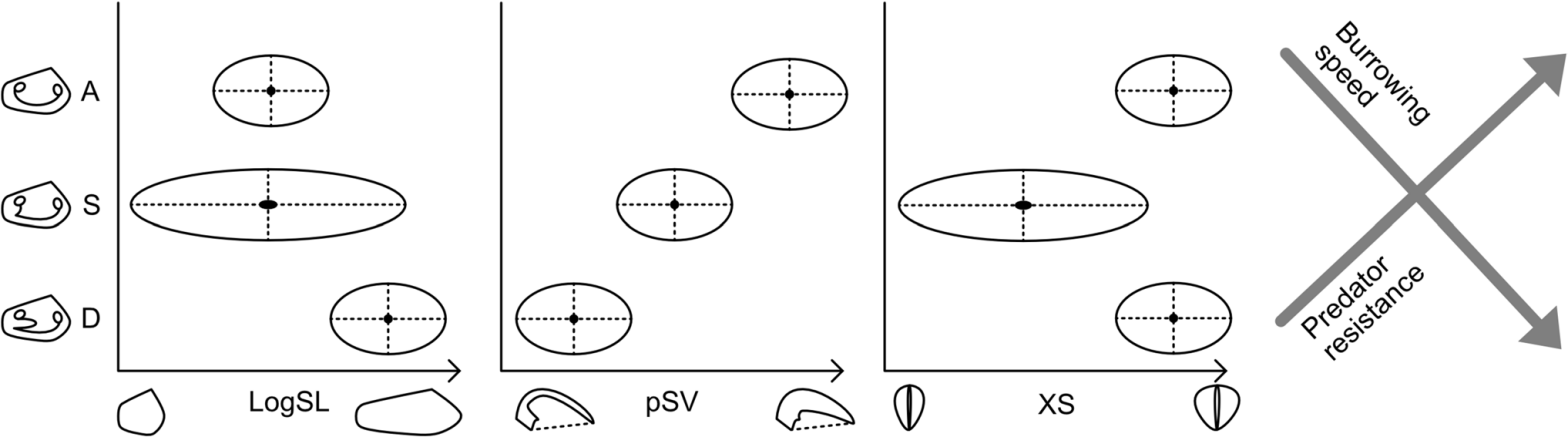
Hypothesized relationships between morphological variables and burial conditions (asiphonate [A], shallow-siphonate [S] and deep-siphonate [D]) and their relationships to burrowing efficiency and predator resistance, as detailed in text. Note that we expect borers to cluster with deep-siphonate taxa and epifauna to cluster with asiphonate taxa for these variables. We expect overall longer shells in deep-siphonate and boring taxa, shorter shells in asiphonate and epifaunal taxa, and greatest variance in shell length for shallow-siphonate taxa. We expect highest pSV in asiphonates and epifauna, and lowest pSV in deep-siphonates and borers. We expect asiphonates, epifauna, borers and deep-siphonates to have similar, wide cross-sections, and shallow-siphonates to have a greater range of cross-sections, generally narrower than the other two groups (see text). Observations from the literature are that burrowing efficiency decreases, but predator resistance increases, as shells become longer, wider, or heavier

The Veneridae and Archiheterodonta provide an opportunity to test the impact that a single trait can have on the diversification and variance of body plans in a group of related animals. By comparing the siphonate Veneridae to the asiphonate Archiheterodonta in terms of morphological traits, we aim to assess what effects, if any, the presence or absence of siphons has had on present-day diversity and disparity, and whether the presence of siphons in Veneridae can be considered a trait key specifically for their prolific diversification, over and above the other traits which also affect their morphology and function, most of which are shared between imparidents and archiheterodonts. Different aspects of shell morphology interact and trade off with each other, and with physics and muscle physiology, in their effects on how the animal interacts with its substrate (Fig. 2). We examine shell length (hereinafter SL), which is relevant to substrate use in terms of both burial depth and burrowing efficiency (Stanley, 1970); the cross-sectional aspect ratio of the shell (hereinafter XS), which affects stability in the substrate and burrowing efficiency (Savazzi & Peiyi, 1992; Trueman, 1966; Vermeij & Dudley, 1985); and proportional shell volume (hereinafter pSV), which captures shell weight and thickness, and thus has multiple downstream effects on predator handling time, stability in the substrate, and burrowing efficiency (Stanley, 1970; Boulding, 1984, Dietl, 2003, Fässler & Kaiser, 2008). We bin burial depth in bivalves into ‘semi-infaunal’, ‘shallow’, and ‘deep’ following the observational work of Stanley (1970) and Kondo (1987) and the ecospace analysis of Mondal & Harries (2016), and we use it here (as ‘asiphonate’ (implying shallow), ‘shallow-siphonate’, and ‘deep-siphonate’) alongside ‘borer’, ‘nestler’, and ‘epifauna’ as categories for consistency with previous (e.g. Edie et al., 2018, Collins & Edie et al., 2019) and future analyses of bivalve ecology.

The bulk of both clades examined here are infaunal. We expect shallow-siphonate bivalves to exhibit a wider range of lengths and cross-sectional aspect ratios than both deep-siphonate and asiphonate bivalves, because the shallow-siphonate group can exploit a greater range of depths in order to evade predators and scour than asiphonates, but are not able to reach depths great enough to completely escape either (as deeper-burrowing taxa have). Thus, they are likely to show a range of morphologies depending on each taxon’s optimal compromise with the predators and wave climate in its habitat. We expect siphonate bivalves to have overall lower pSV values than asiphonate bivalves, as they may be less dependent on shell thickness as a key protection against predators. We expect endolithic borers to be similar in form to deep-siphonates, and epifaunal taxa to be similar in form to asiphonates, due to similar levels of proximity to wave climate and predation. These predictions are summarized in Fig. 2.

## Materials and Methods

### Specimens

At least one representative adult or near-adult specimen (i.e. within adult size range reported in the literature) was sampled from museum collections (Supplementary Table 1) for 12 astartid, 72 carditid, and 40 crassatellid species (total: 124 species), together making up the archiheterodont clade for the purposes of this study, plus 126 venerid species, each representing a genus. These specimens represent 62% of the extant archiheterodont genera, and 90% of the genera in the more diverse venerids. Genera which are not represented were omitted due to lack of available specimens. The data for each specimen are found in Supplementary Table 1. For consistency, the left valve of every specimen was chosen for scanning; where no left valve was available, the right valve was scanned and operationally mirrored to approximate a left valve. This is appropriate since all taxa in our analyses are equivalve, and the valves of equivalve taxa are fundamentally mirror images of each other except for the interlocking elements of the hinge, the exact configuration of which is not considered in our analyses.

### Capturing 3D Morphologies

Shells were micro-CT scanned with the University of Chicago Paleo-CT facility’s GE v|tome|x scanner, using the 240 kV micro-CT tube. Scan resolutions vary between 7.8 and 84.1 microns per voxel (mean = 41.3 microns per voxel). The voxel data from these scans was used to make watertight, isosurface triangular mesh data using the methods of Collins and Edie et al. (2019): briefly, surfaces were fit to the voxel data from the CT scans and output as ‘.stl’ mesh files in VG StudioMax. Any isolated parts, boreholes or debris were digitally removed (e.g. sand, epibionts, worm/sponge borings) and surfaces were made manifold prior to analyses in Autodesk Meshmixer, manually and with extensive checking by the authors. Meshes can be accessed from Data Dryad (Supplementary File 1).

### Morphological Features

Measurements of morphological features were taken on mesh data that had been aligned to the hinge axis of each individual (see Edie et al., 2022 for details).

### Proportional Shell Volume

Proportional shell volume (pSV) is calculated by dividing the total shell volume of a specimen by the quantity of its total shell volume plus its total internal volume (see diagrams in Fig. 2). Shell and internal volumes of specimens were calculated in this analysis using the “shell proportion” methods of Collins and Edie et al. (2019); in brief, the volume of shell material produced by the animal is calculated as the sum of signed tetrahedra volumes across the surface mesh. The soft-tissue + water volume (‘internal volume’) of the animal is then found by centering the mesh surface of just the shell interior on the origin, and taking the sum of signed tetrahedra volumes. The shell volume and internal volumes are each multiplied by two to calculate the total shell volume (TSV) and total internal volume (TIV). Shell proportion (pSV) is then calculated as TSV/(TSV + TIV).

### Shell Length

*Shell length* (SL) is calculated as the dimension of the bounding box of the shell valve that aligns with the anterior–posterior axis (Fig. 1). For the purposes of comparison in the rarefaction analysis of disparity versus diversity (see section on Analysis below), we also use shell length for all archiheterodont and venerid species, as reported in the literature (Huang et al., 2023).

### Cross-Sectional Aspect Ratio

*Cross-sectional aspect ratio* (XS) is calculated as the ratio of shell width to height. Shell width is the dimension of the bounding box perpendicular to the commissural plane (Fig. 1), which we doubled to produce the shell width of a complete individual. Shell height is the dimension of the bounding box orthogonal to shell length (Fig. 1). A specimen whose width was equal to its height would have an aspect ratio of 1, if width is greater than height the aspect ratio is > 1, and if height is greater than width the aspect ratio is < 1.

### Functional Groups

Bivalve genera were assigned to the four-dimensional functional groups used by Collins and Edie et al. (2019), in which the relative burrowing depth category is informed by previous functional analyses (i.e. Kondo, 1987; Stanley, 1970). Because this project focuses on specific lineages, and by design aims to hold as many functional attributes constant as possible, only one of the four functional axes, *substratum use* (also termed *substrate* herein), shows any variability in our dataset, and we use only this functional axis in our analyses.

To assign siphonate taxa as either deep vs shallow burrowing, we consider the burial depth of the posterior margin, and use a cutoff of 30 mm posterior burial depth–deeper than 30 mm is considered ‘deep’, and above it is considered ‘shallow’ (as in Stanley, 1970). We assume minimal siphon extension for venerids, in keeping with Kondo’s (1987) observations and general relationship of sinus depth to siphon length, and so species under 50 mm adult shell length are all considered shallow-burrowing. Species longer than 50 mm had their pallial sinus depth checked—if the sinus extends to ¾ of a 50 mm shell length, then the siphon could be capable of being 35 mm long, suggesting that the taxon could live deeper than 30 mm below the surface.

### Phylogenetic Hypothesis

To account for the influence that shared ancestry may have on our analyses, we constructed a time-calibrated topology of genera included in this study using publicly available molecular data from GenBank, published morphological phylogenies, cladistics, and the family’s fossil record, following the method of Edie et al. (2022). The venerid portion of the phylogeny and fossil genus ages presented here are derived from that tree, with taxa excluded if they were not in our morphological dataset. The archiheterodonts are poorly sampled in terms of molecular data (about 28 out of 265 species; González & Giribet, 2015); taxa from the archiheterodont families were grafted onto the molecular topology using published morphological phylogenies or cladistic relationships [as in the approach of Soul and Friedman (2015) and Edie et al. (2022)). We time-scaled the phylogeny using treePL (Smith & O’Meara, 2012) with the first known stratigraphic occurrence for all genera following a budding model of evolution, where the younger of the two daughter lineages dates the split (Crouch et al., 2021) (archiheterodont fossil ages determined from an extensive literature search [Supplementary Table 1]).

### Analysis

We examined how SL, XS, and pSV differ between families, presence/absence of siphons, and substratum-use functional groups. We tested for covariance between morphological traits using the function pairs.panels() (from R package ‘psych’) which provides scatterplots and reports Pearson’s correlation coefficient (*r*). We performed linear model fits using the function lm.rrpp() and then analysis of variance (ANOVA) using the function anova() in R package ‘RRPP’ (Collyer & Adams, 2018) to test for statistically significant relationships between morphological traits and either family membership or substratum use, accounting for shared ancestry using a variance–covariance matrix computed from the phylogenetic tree. To assess the relationship between clade diversity and disparity in the morphological variables, we also performed rarefaction, where the dataset was resampled to sample sizes ranging from 5 specimens to the full dataset (Astartidae: 12, Carditidae: 72, Crassatellidae: 40, Veneridae: 126), for the calculation of variance and range of the morphological variables. In order to have some idea of the effect of taxon and specimen selection upon the pattern, we include a comparative dataset consisting of the maximum reported shell length (the only metric used in this paper that is widely reported elsewhere) from the literature of all species in the families analyzed here (Huang et al., 2023). Each sample size was repeated 1000 times so that a median variance and range for the variable at a sample size of *n* could be calculated. All code used to generate results and plots is in Supplementary Material.

## Results

### Proportional Shell Volume

**Fig. 3:**
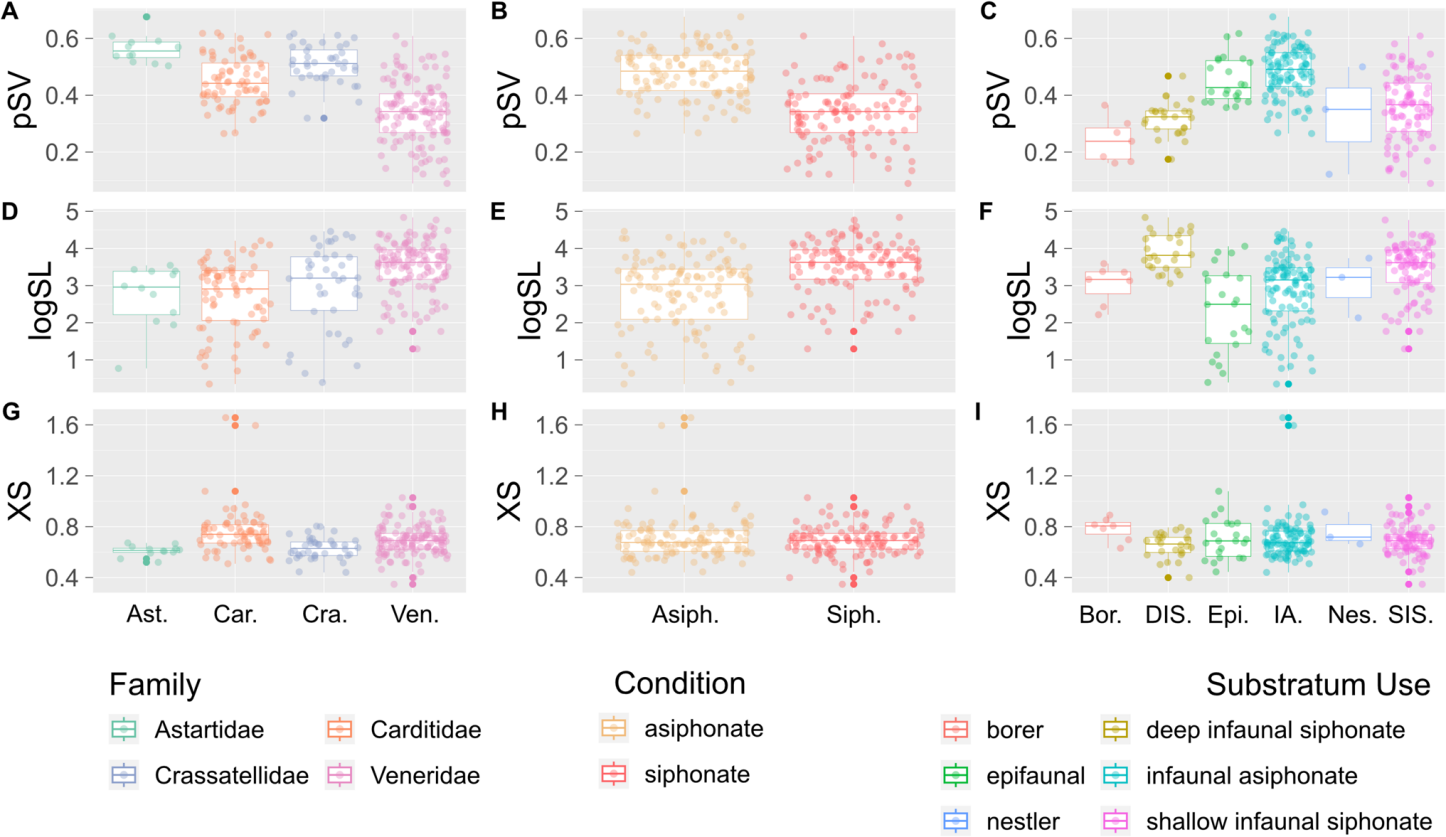
Boxplots of the three morphological traits (proportional shell volume (pSV: **A**–**C**), shell length (logSL: **D**–**F**), and cross-sectional area (XS: **G**–**I**)), partitioned by family (**A**, **D**, **G**), siphonate condition (**B**, **E**, **H**), and substratum use (**C**, **F**, **I**) (Almost all archiheterodonts are infaunal asiphonate—a small number of carditids are epifaunal. All other substratum uses are represented by members of the Veneridae). Each boxplot has the individual points for each group plotted as a jitter over it

**Fig. 4:**
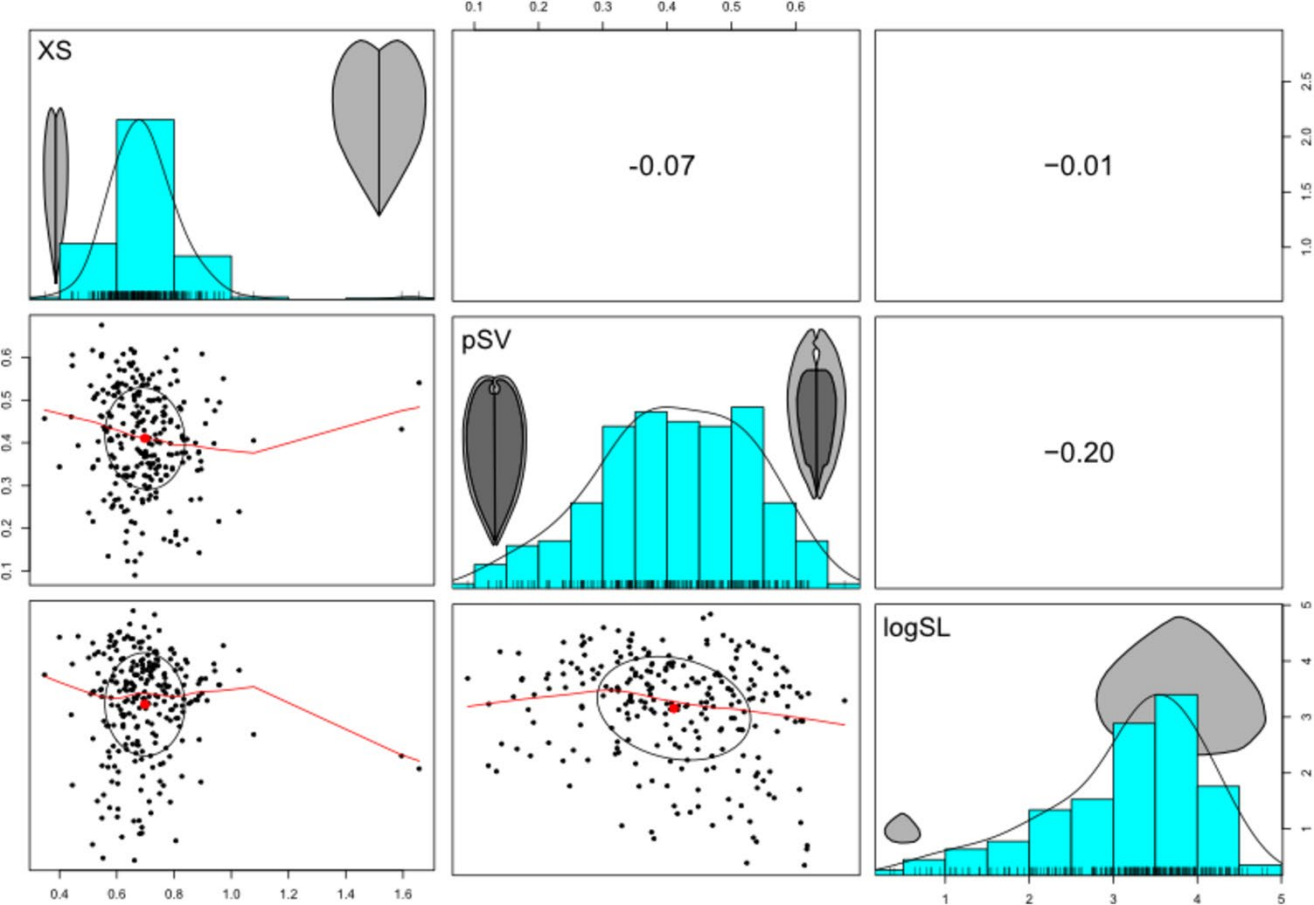
A scatterplot matrix with histograms, showing the correlations among the three morphological variables (XS = shell cross-sectional aspect ratio; pSV = proportional shell volume; logSL = logarithm of shell length), and their linear Pearson correlation coefficient values. The ellipses encompass 95% confidence intervals and their shape indicates the extent of correlation (the narrower the ellipse, the stronger the correlation); red lines are LOESS smoothed fits. Schematics illustrate the extreme states of each variable but are not representative of specific taxa

**Fig. 5:**
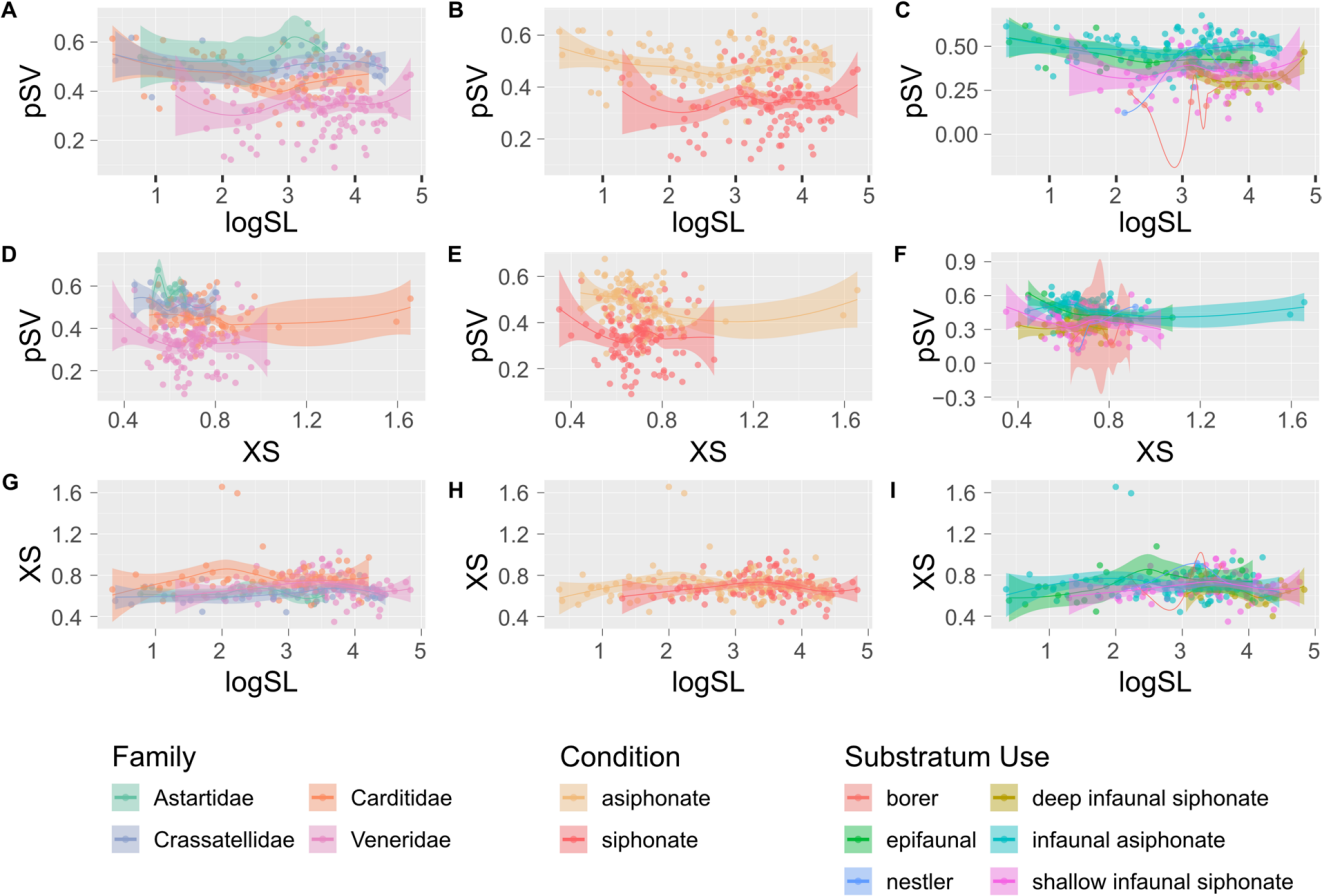
Scatterplots showing the relationship of proportional shell volume with shell length (**A**–**C**) and cross section (**D**–**F**), and the relationship of cross section with shell length (**G**–**I**), partitioned according to family, clade, or substratum use. Points are specimens, solid lines are LOESS curves with 95% confidence intervals (shaded bands), calculated using the R function *geom_smooth()* from the package *ggplot2*. Small groups (borers, nestlers) do not have sufficient numbers to calculate a confidence interval and are shown as LOESS curves only

Epifaunal bivalves have a higher pSV (are thicker-shelled) than the four other venerid-only substratum groups (i.e. borer, deep infaunal siphonate, nestler, and shallow infaunal siphonate groups), and infaunal asiphonate bivalves have the highest pSV overall; both of these substratum groups are composed exclusively of archiheterodonts (Fig. 3C). Among the venerids, the borers and deep infaunal siphonates have low pSV (are thin-shelled), while nestlers and shallow infaunal siphonates show a broad range of values. pSV has only a very weak linear correlation with SL or XS; r = − 0.20 and 0.11 respectively (Fig. 4). As SL and XS increase, pSV remains generally constant, partitioned by clade or substratum group (Fig. 5).

### Shell Length

There is considerable overlap between families and substratum groups in logSL (Fig. 3D, F), although venerids tend to be larger than the three archiheterodont families (seen most clearly in Fig. 3D). Similarly, the six substratum-use groups (Fig. 3C) show much overlap; the exclusively venerid deep and shallow infaunal siphonate groups exhibit the largest median sizes. There is a striking difference between the asiphonate and siphonate conditions (Fig. 3E).

### Cross-Sectional Aspect Ratio

There is also considerable overlap in XS among family and substratum groups (Fig. 3G, I). Venerids reach more extreme blade-like shapes than archiheterodonts, particularly the shallow-infaunal suspension feeding venerids.

### Phylogenetic ANOVA

The three morphological variables analyzed—logSL, XS, and pSV—all show strong phylogenetic signal (p < 0.0001) as calculated using both Pagel’s λ and Blomberg’s K (Supplementary Table 2). We report here only on the three most complex models used in the phylogenetic ANOVA, but the results of simpler models examining different combinations of variables are also available in Supplementary Table 3.

We find that pSV has a significant relationship with both substratum group (p = 0.001) and family (p = 0.016) (Table 2), whereas logSL is only significantly correlated with substratum group (p = 0.001) (Table 3) and XS is only significantly correlated with family (p = 0.003) (Table 4).

**Table 2:** Phylogenetic ANOVA results for the model (pSV ~ substrate + logSL + XS + family)

	Df	SS	MS	Rsq	F	Z	Pr(> F)	Sig
Substrate	5	0.6171	0.123429	0.17390	14.3266	6.2910	0.001	***
logSL	1	0.0001	0.000053	0.00001	0.0062	− 1.5368	0.932	
XS	1	0.0012	0.001186	0.00033	0.1376	− 0.6098	0.715	
Family	2	0.1447	0.072362	0.04078	8.3992	3.2895	0.001	**
Residuals	240	2.0677	0.008615	0.58262				
Total	249	3.5489						

See Supplementary Material for full model parameters and the results of other models mentioned in text. pSV has a strong relationship with both family and substratum use but not log SL or XS

**Table 3:** Phylogenetic ANOVA results for the model (logSL ~ substrate + XS + pSV + family)

	Df	SS	MS	Rsq	F	Z	Pr(> F)	Signif
Substrate	5	18.757	3.7515	0.08768	5.4038	3.5978	0.001	***
propSV	1	0.652	0.6521	0.00305	0.9393	0.4466	0.345	
XS	1	0.004	0.0043	0.00002	0.0062	− 1.5974	0.938	
Family	2	2.288	1.1440	0.01070	1.6478	0.8548	0.204	
Residuals	240	166.614	0.6942	0.77886				
Total	249	213.920						

See Supplementary Material for full model parameters and the results of other models mentioned in text. Log SL has a strong relationship with substratum use, but not pSV, XS, or family

**Table 4:** Phylogenetic ANOVA results for the model (XS ~ substrate + logSL + pSV + family)

	Df	SS	MS	Rsq	F	Z	Pr(> F)	Signif
Substrate	5	0.1636	0.032728	0.03359	1.8966	1.1949	0.117	
pSV	1	0.0162	0.016208	0.00333	0.9393	0.4718	0.345	
logSL	1	0.0024	0.002375	0.00049	0.1376	− 0.5952	0.706	
Family	2	0.5319	0.265926	0.10917	15.4106	3.8673	0.001	***
Residuals	240	4.1415	0.017256	0.85007				
Total	249	4.8719						

See Supplementary Material for full model parameters and the results of other models mentioned in text. XS has a strong relationship with family, but not substratum use, pSV, or log SL

### Disparity Versus Diversity

**Fig. 6:**
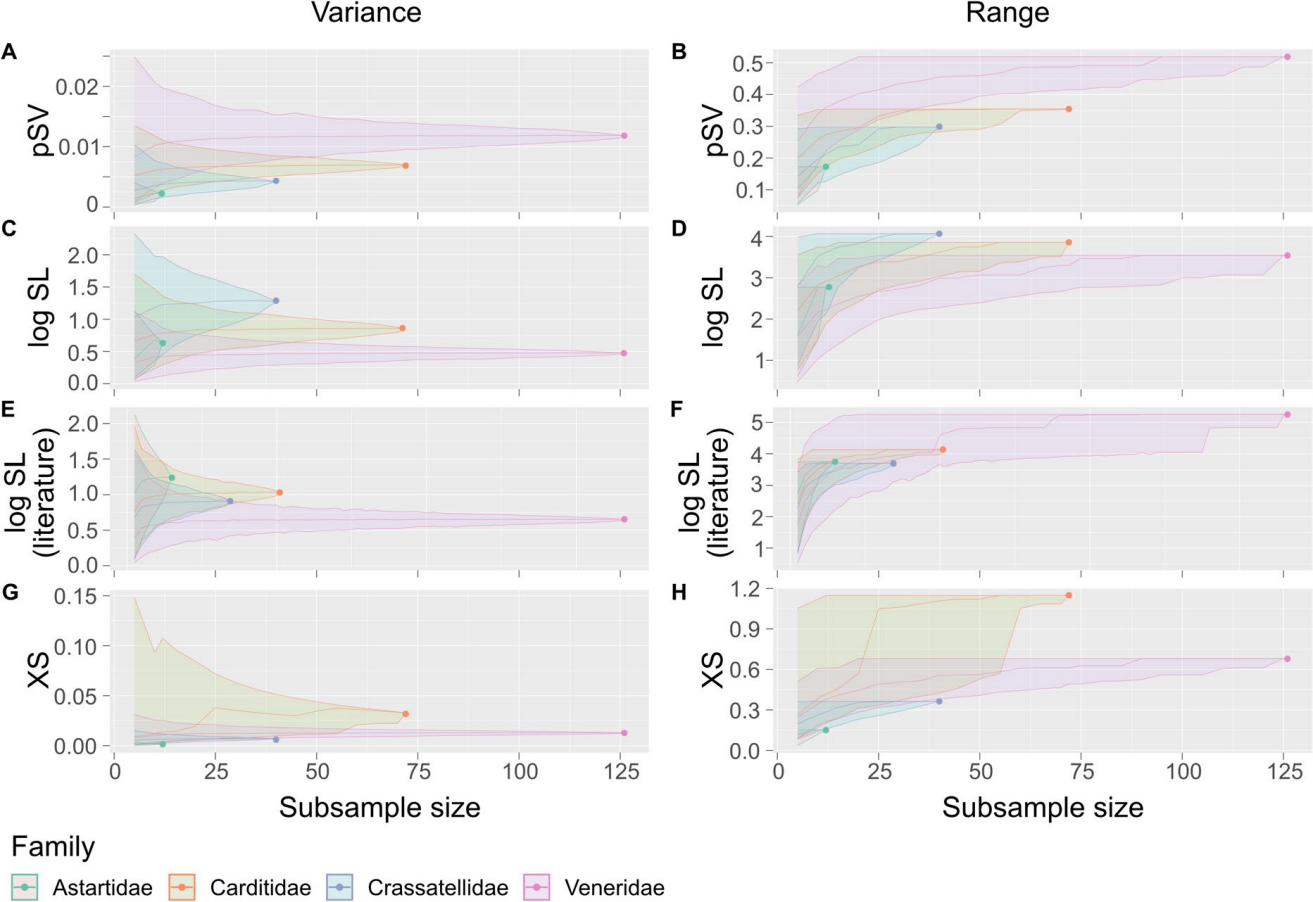
Rarefaction curves for the median disparity (two different metrics: variance and range) and 95% confidence intervals (shaded area) of pSV of the species analyzed here (**A**, **B**); logSL of the species analyzed here using CT, (**C**, **D**); logSL calculated from a literature compilation by Huang et al. (2023) of body lengths for all species (**E**, **F**); and XS (**G**, **H**) of the species analyzed here. Measured disparity for the full sample of each family is shown by a point at the far right of the confidence envelope

Does disparity in the morphological variables rise simply as a function of taxonomic diversity? Fig. 6 shows rarefaction curves for pSV, XS and logSL (using CT data for all three variables, and additional logSL data from the literature compilation of Huang et al. (2023)), using two measures of disparity: the variance and the cumulative range. The subsample sizes at which the confidence envelopes diverge for any two families indicate the sample size at which the disparity of the morphological variable of interest is differentiable between those two families.

For pSV, the more speciose the family, the higher the disparity (by either metric) (Fig. 6A, B), but the disparity measures actually stabilize at smaller sample sizes than the final total. The sample size at which the confidence intervals diverge between families indicates roughly how many species are needed to detect the disparity difference between them: for Astartidae versus Veneridae, using the variance, the threshold is just 8 species. Crassatellids are differentiable from venerids at a sample size of 20; carditids are differentiable at a sample size of 45. Using the cumulative range measure (panels B, D), we can see that the true range value (where the curve flattens) is actually attained before the sample size equals the full sample for all for families: 10 species for astartids, 30 for crassatellids, 40 for carditids and 90 for venerids. These thresholds indicate among-family differences in disparity for pSV, even at low sample sizes.

For logSL, the disparity is not related to sample size when measured by variance: the highly diverse Veneridae have much lower disparity than the lower-diversity archiheterodonts, both in the CT dataset used for analyses herein (Fig. 6C, D) and in a comprehensive body-size dataset for the families analyzed here (Fig. 6E, F, calculated from the literature compilation also used in Huang et al., 2023). The archiheterodont families do have different rarefaction curves for logSL when using the CT dataset versus the literature dataset, but this derives from the different sample sizes. The pattern holds, however, that no disparity difference is detectable between families using shell length (from either method) until sample size reaches at least 38. Venerid shell length variance is always the lowest of all families, but they have the greatest range when the full dataset from the literature is used, which is not recovered from our CT dataset, indicating that the incompleteness of the CT dataset does have an effect on the results. As neither XS nor pSV are correlated with logSL (Figs. 4, 5), we have no reason to suspect that the results for those two variables are likely to be similarly affected, but additional reporting of height, width, and volume information for other taxa would of course be a valuable addition for future analyses.

Disparity in XS is to some extent correlated to diversity: disparity (measured both ways) rises steadily with sample size for astartids, crassatellids and venerids, all of which overlap confidence intervals for both metrics. Of the archiheterodonts, carditids show the highest disparity.

## Discussion

### Impact of Siphons on Diversity and Disparity

Boulding (1984, p. 202) in her study of crab predation on bivalves says, “The bivalve shell is obviously an example of morphological compromises in evolution,” a masterful summary of the situation of interacting traits that contribute to bivalve shell form. Many shared traits—among all bivalves or between our two focal groups—contribute to the variety of ways that bivalves live, and our aim here has been to assess if a specific, derived trait, i.e., siphons, can be viewed as pivotal not just to individual function, but to taxon diversification. Archiheterodonts and venerids are in many ways remarkably similar, including in shell form. But even though the oldest archiheterodonts (*Cypricardella* and *Prosocoelogeton*) originated 250 million years before the oldest venerid (*Austrocardilanx*) (Crouch et al., 2021), and archiheterodonts survived the end-Cretaceous extinction with more genera than venerids (Edie et al., 2018), archiheterodont families have failed to diversify as widely as the Veneridae.

Our results suggest that the groups of bivalves studied here have ‘tuned’ their inherited size and shape in order to have specific characteristics for burrowing efficiency, stability in the sediment, and predation resistance (Fig. 5). However, it is the presence or absence of siphons that ultimately controls the possible range of depths in the substratum that a bivalve can exploit. If a key trait is defined as one that allows the organism to interact with the environment in a new way (de Queiroz, 2002; Jablonski, 2022; Miller, 1949; Miller & Stroud, 2021; Rabosky, 2017), then bivalve siphons are surely a textbook example. But has the siphonate condition led to increased diversity and disparity for bivalves that exhibit it? And conversely, does the lack of siphons restrict asiphonate taxa to low diversification and disparification?

Diversification is easy to address, and has been long-known to be high in siphonate bivalves (e.g., Stanley, 1968). Of the dominantly infaunal heterodont bivalve taxa, the number of siphonate genera and species (Anomalodesmata plus Imparidentia) far outstrip those in asiphonate clades (Palaeoheterodonta plus Archiheterodonta) (Table 1). At the family level, the Veneridae is the most speciose group of all extant Bivalvia (and they occupy five different functional groups, a number matched only by Mytilidae and Myidae, both of which are also siphonate).

Disparification is more difficult to quantify. We find different behaviors of our three morphological variables with respect to increasing taxonomic diversity. Disparity of XS increases with diversity at at a similar rate in three of the four families, but at a much greater rate for Carditidae. Disparity of pSV does increase with taxonomic diversity, but not linearly: as illustrated by Fig. 6, each family has a characteristic level of disparity that it reaches before the full diversity of the sample is included in the calculation. In contrast, disparity of logSL is not correlated with taxonomic diversity: the most disparate family in terms of variance in shell length is the low-diversity Crassatellidae, and the hyper-diverse Veneridae have the second lowest logSL range and lowest logSL variance in the micro-CT dataset, because venerid species are so densely packed within the range of sampled sizes. When additional species in the literature are included (Fig. 6E, compared to C, and F compared to D), the smaller families swap places relative to each other, because more taxa are included, and the Veneridae logSL range exceeds each of the archiheterodont families, but crucially, its variance remains low. This shows that, as species accumulate, those species are not always pushing the morphological envelope: there is redundancy, or ‘packing,’ in the morphospace of some variables, but the venerids ultimately do achieve the greatest range of logSL values.

### Morphological Adaptations to Substrata

Each family has a characteristic “shape” (in terms of logSL and XS) that interacts with pSV to “tune” species to their substrate. Phylogenetic signal results (Supplementary Table 2) indicate that there are family-level effects on form. Longer shells, and shells with wide cross-sections, are not necessarily thicker-shelled, meaning that bivalves of the same length or cross-section may differ significantly in pSV: archiheterodonts have thicker shells than venerids for a given ‘footprint’ against their substratum (the cross-sectional area and length) (Fig. 5B, E).

We hypothesize, based on observations from literature (e.g. Stanley, 1968, 1970; Seed & Brown, 1978 and others cited below) that this tuning may occur in a number of ways. Large, wide bivalves have slower burrowing efficiencies, but can lighten themselves and thus burrow faster by reducing pSV. Small, narrow bivalves that live at the sediment–water interface can combat predation and wave action with thicker, and thus heavier, shells. Siphonate taxa also need to contend with non-lethal ‘nipping’ of their siphons by predators, and they may try to protect themselves with additional shell ornamentation (as in *Hysteroconcha lupanaria* [see Stanley, 2015a]) or simply put up with the nipping and have to modify behavior (e.g. burrowing more shallowly to compensate for a shortened siphon [Cledón & Nuñez, 2010], requiring a shell form that is able to cope with a shallower burial depth) or expend extra energy and resource to regrow siphonal tissue, which is observed to have knock-on effects on growth and reproduction (Tomiyama & Omori, 2007), and thus, shell form.

pSV varies with burial depth, as resistance to predation and scour becomes less relevant with depth (Stanley, 1988). Epifaunal species and asiphonate species that live closest to the sediment surface (i.e. archiheterodonts) have thicker shells than shallow siphonate infauna, which can live deeper in the sediment, and deep-infaunal taxa have the thinnest shells, along with borers. There is a clear pattern of pSV increase as burrowing depth decreases (Fig. 3C); it is possible that epifaunal taxa are thinner-shelled than infaunal asiphonate bivalves due to differing strategies for predation response. Epifaunal bivalves in our study group may analogously be accelerating their growth to reach a large size, while infaunal asiphonate bivalves might put more energy into producing a thick shell since they will be vulnerable to predators no matter their size. Note that pSV describes a largely flat line for all groups when plotted against logSL (Fig. 5A–C)—regardless of shell size, the relative thickness remains constant. Freshwater mussels also reflect dual solutions to a similar problem: species occurring in rivers with high flow rate have relatively thicker shells, promoting stability in the substratum, compared to those in lower flow rate rivers, which have thinner shells that are more efficient for reburrowing following disturbances (Keogh et al. 2024). Disambiguating the reasons a bivalve may have a thinner or thicker shell will require direct experimentation, and more observational studies of behaviour and anatomy, but it is possible to develop hypotheses for this kind of testing using data such as we present herein.

### Siphons as a Key Trait

Siphonate heterodonts unambiguously outstrip asiphonate heterodonts in taxonomic diversity (Table 1). Siphonate bivalves today exhibit varying degrees of disparity and diversity; the Veneridae do represent a resounding success story, but their close cousins, the Arcticidae, have been reduced to a single extant species. Presumably, ecology and other aspects of morphology also have a hand to play in the success of any given subclade. However, despite the varying fortunes through time of the individual families, there are four times as many fossil siphonate genera, and ten times as many extant siphonate genera, than in the asiphonate groups.

In morphological terms, the analyses reported here show that pSV is more disparate in venerids (in both variance and range) than in archiheterodonts. The fact that pSV does not covary with shell length or cross-section, and that the differences in pSV between families are so striking, suggests strongly that this is a character with phylogenetic affinity in the archiheterodonts. Further, given the inverse relationship of pSV with depth of burial, and that venerids are also more diverse than archiheterodonts, it is likely that siphons have opened up additional modes of life for venerids, allowing increased diversification and disparification (of size and shell thickness) compared to archiheterodonts. Conversely, the lack of siphons in archiheterodonts may constitute an adaptive limitation as these taxa are less disparate in shell thickness and less diverse both functionally and taxonomically than venerids. The asiphonate Archiheterodonta do generally have higher disparity in logSL than venerids, as measured by variance, possibly reflecting their low diversification rate: while there may be a wide variety of viable shell lengths for an infaunal bivalve, there are simply fewer taxa in the group, and thus a lower density in the size morphospace, and for the taxa studied here, the largest sizes are associated with the siphonate condition.

Infaunal asiphonates are the most shallowly-buried group on average, but some shallow infaunal siphonate burrowers also live very close to the sediment–water interface. We hypothesize that these shallow infaunal siphonate taxa have thinner shells than archiheterodonts living at the same depth precisely because they have siphons. This trait requires space inside the shell for siphons to be accommodated when retracted, but enables venerids to invest energy into building soft tissues to feed more efficiently whereas archiheterodonts are forced to put more energy into growing a protective thick shell.

Siphons allow venerids to exploit deeper burial in the sediment, or to bore deeply into rocks, leading to a tiering of taxa at different depths and partitioning of the ecological landscape. Venerids are more diverse than archiheterodonts in terms of species numbers, pSV, and substratum use groups, and show a broader range of sizes. The asiphonate archiheterodonts compensate for the pressures of life at the sediment–water interface via greater pSV, but this adaptation evidently has not compensated archiheterodont for the lack of siphons, and thus they show low diversification. Based on the findings documented herein, we therefore argue that siphons do constitute a key adaptive trait for bivalves.

### Placing Siphons in an Evolutionary Context

There are many documented examples of key traits in evolution, though definitions and study measures vary. Some, like the beetle *Stenus* with its specialized protruding labium for capturing prey, are similar to siphons in bivalves in that the key trait promotes high diversity without correspondingly high morphological disparity (Minelli, 2016; Puthz, 2010). In other instances, a key trait promotes a large increase in both taxonomic diversity and morphological disparity, as the cyathium did for the plant group *Euphorbia,* allowing a shift from wind to insect pollination (Horn et al., 2012; Minelli, 2016). Further, there are examples of high morphological disparity early in a clade’s existence when taxonomic diversity is still low (such as in Cambrian marine arthropods and Cenozoic ungulates) (Foote, 1997). The vast majority of key-innovation studies have used species richness or diversification rate in their criteria (Miller et al., 2023); more analyses are needed that examine taxonomic diversity in combination with other scales, such as morphological disparity and functional diversity.

In the case of bivalve siphons and differential diversification, it is likely that differing reproductive strategies are also an important factor, with archiheterodonts generally having low fecundity, with low-dispersal, often brooded larvae, compared to venerids, which are heavily dominated by high-fecundity, high-dispersal development (Malchus & Sartori, 2013), potentially a critical advantage for prolific low-latitude diversification in the face of intensifying predation and other biotic interactions (e.g. Krug et al., 2007; Vermeij, 1987). Additional information on reproductive modes for a wider swath of taxa in these families would help to further isolate the effect of siphons on taxonomic diversification.

## Conclusions

Archiheterodonts are largely similar to venerids in their anatomy and physiology but lack siphons—they originated earlier, giving them more time to accumulate diversity and disparity, and fared better in the end-Cretaceous mass extinction 66 Myr ago, and so should have been poised to diversify as vigorously as the venerids. But the extant asiphonate archiheterodonts have lower diversity, lower morphological disparity, and lower functional disparity than the siphonate venerids. Siphons thus appear to be a key adaptive trait in venerids, promoting their diversification. The trait’s impact on the different macroevolutionary currencies was non-linear, however, with a stronger effect on venerid taxonomic diversity than morphological disparity, causing denser morphospace packing—and thus reduced variance-based disparity than in archiheterodonts. This supports the body of literature finding that key traits can have nuanced and varied impacts on clade radiation in different evolutionary contexts.

## Supplementary Information

Below is the link to the electronic supplementary material.

Supplementary file1 (R 31 KB)

Supplementary file2 (NEX 20 KB)

Supplementary file3 (CSV 59 KB)

Supplementary file4 (CSV 16 KB)

Supplementary file5 (XLSX 68 KB)

Supplementary file6 (CSV 1 KB)

Supplementary file7 (CSV 2 KB)

## Data Availability

Metadata, taxonomic and phylogenetic details for all specimens, code for all analyses and figures, and additional tables of results for phylogenetic signal and ANOVA are available as an electronic supplement. Mesh models are available on Figshare (10.6084/m9.figshare.27890193.v1).
